# A Cross-Sectional Observational Study of Non-alcoholic Fatty Liver Disease Assessed by Fibrosis Index in Patients With Type 2 Diabetes Mellitus Presenting to a Tertiary Care Centre in Southwestern Maharashtra

**DOI:** 10.7759/cureus.109686

**Published:** 2026-05-26

**Authors:** Pratik U Patil, Aparna P Patange, Swati Aundhakar, Udaysinh V Patil

**Affiliations:** 1 Department of Medicine, Krishna Institute of Medical Sciences, Krishna Vishwa Vidyapeeth (Deemed to be University), Karad, IND; 2 Department of Gastroenterology, Krishna Institute of Medical Sciences, Krishna Vishwa Vidyapeeth (Deemed to be University), Karad, IND

**Keywords:** diagnostic accuracy, fib-4 index, liver fibrosis, non-alcoholic fatty liver disease, non-invasive screening, type 2 diabetes mellitus, ultrasound elastography

## Abstract

Background: Non-alcoholic fatty liver disease (NAFLD) is highly prevalent among patients with type 2 diabetes mellitus (T2DM) and is associated with progressive fibrosis and adverse clinical outcomes. Early identification of fibrosis using non-invasive methods is essential. This study aimed to evaluate the diagnostic utility of the Fibrosis-4 (FIB-4) index and compare it with ultrasound elastography in detecting liver fibrosis.

Methods: A cross-sectional observational study was conducted in a tertiary care center and included 150 patients with T2DM. Demographic, clinical, and laboratory parameters were recorded. The FIB-4 score was calculated using age, aspartate aminotransferase (AST), alanine aminotransferase (ALT), and platelet count. Liver stiffness was assessed using ultrasound elastography, and fibrosis was staged from F0 to F4. Statistical analysis included correlation and receiver operating characteristic (ROC) curve analysis.

Results: Out of 150 patients, 110 (73.3%) were found to have NAFLD on ultrasound. Fibrosis staging revealed that the majority of patients were in stage F2 (52.0%), followed by F3 (27.3%), F0 (13.3%), F1 (4.7%), and F4 (2.7%). The mean liver stiffness was 8.97 ± 2.50 kPa. A strong positive correlation was observed between FIB-4 score and liver stiffness (ρ = 0.804, p < 0.001). AST showed a strong positive correlation with liver stiffness (r = 0.744, p < 0.001), whereas platelet count demonstrated a moderate negative correlation (r = -0.472, p < 0.001). The diagnostic performance of FIB-4 for detecting significant fibrosis was excellent, with an area under the ROC curve of 0.951 (95% CI: 0.918-0.983).

Conclusions: NAFLD and significant liver fibrosis are highly prevalent in patients with T2DM. The FIB-4 index demonstrates strong correlation with elastography and excellent diagnostic accuracy, making it a reliable, cost-effective, non-invasive screening tool for liver fibrosis.

## Introduction

Non-alcoholic fatty liver disease (NAFLD) has emerged as the leading cause of chronic liver disease worldwide, paralleling the rising prevalence of metabolic syndrome and type 2 diabetes mellitus (T2DM) [1,2]. It encompasses a wide spectrum of hepatic pathology ranging from simple steatosis to non-alcoholic steatohepatitis (NASH), progressive fibrosis, cirrhosis, and ultimately hepatocellular carcinoma [3]. Among these stages, liver fibrosis represents the most critical prognostic factor, as it is strongly associated with liver-related morbidity, mortality, and overall survival [4]. Progression of fibrosis is often insidious and may remain undetected until advanced stages, making early identification crucial for preventing irreversible liver damage [5].

Patients with T2DM are particularly vulnerable to NAFLD due to underlying insulin resistance, chronic low-grade inflammation, oxidative stress, and altered lipid metabolism [6,7]. Epidemiological studies suggest that up to 70%-80% of individuals with T2DM have underlying NAFLD, many of whom remain asymptomatic until advanced stages of disease [8,9]. This silent progression underscores the importance of early detection and risk stratification of liver fibrosis in this high-risk population. Moreover, NAFLD in diabetic individuals is associated with an increased risk of cardiovascular disease and overall mortality, further highlighting its clinical significance [10].

Although liver biopsy remains the gold standard for the assessment of hepatic fibrosis, its routine use is limited by its invasive nature, potential complications, sampling variability, and cost [11]. Consequently, there has been a growing emphasis on non-invasive modalities for fibrosis assessment. Among these, serum-based indices and imaging techniques have gained widespread clinical acceptance.

The Fibrosis-4 (FIB-4) index is a simple, non-invasive scoring system derived from readily available clinical and laboratory parameters, including age, aspartate aminotransferase (AST), alanine aminotransferase (ALT), and platelet count [12,13]. It has been validated across multiple populations as an effective tool for identifying patients at risk of advanced fibrosis. On the other hand, ultrasound elastography provides a direct, quantitative assessment of liver stiffness, which correlates with the degree of fibrosis and has become an important tool in routine clinical practice [14]. Given the increasing burden of NAFLD in patients with T2DM and the need for accessible, reliable, and cost-effective diagnostic tools, this study was conducted to evaluate the utility of the FIB-4 index in detecting liver fibrosis and to compare its performance with ultrasound elastography in a tertiary care setting.

## Materials and methods

Study design and setting

This was a hospital-based, cross-sectional observational study conducted at a tertiary care teaching hospital. The study was carried out over a defined study period after obtaining approval from the Institutional Ethics Committee, Krishna Vishwa Vidyapeeth (Deemed to be University), Karad, India (approval no.: KVV/IEC/05/2024; date: 17-04-2024).

Study population

A total of 150 patients diagnosed with type 2 diabetes mellitus (T2DM) were enrolled in the study. Patients attending the outpatient department or admitted to the hospital were screened and recruited based on predefined inclusion and exclusion criteria.

Inclusion criteria

The study included adult patients aged ≥18 years with a confirmed diagnosis of type 2 diabetes mellitus (T2DM).

Exclusion criteria

Patients with alcoholic liver disease were excluded from the study, including males consuming more than 60 g of alcohol per day and females consuming more than 40 g of alcohol per day. Patients with viral hepatitis, including hepatitis B and hepatitis C infection, were also excluded. Additionally, patients with decompensated liver cirrhosis and those unwilling to provide informed consent for participation in the study were excluded from the analysis.

Clinical and laboratory assessment

Detailed clinical history and examination were performed for all patients. Laboratory investigations included liver function tests (aspartate aminotransferase (AST), alanine aminotransferase (ALT)) and platelet count. All investigations were performed using standard laboratory methods.

Calculation of FIB-4 index

The Fibrosis-4 (FIB-4) index was calculated for each patient using the standard formula:

\[ \text{FIB-4} = \frac{\text{age} \times \text{AST}}{\text{platelet count} \times \sqrt{\text{ALT}}} \]

The calculated values were used to assess the likelihood of liver fibrosis.

Imaging assessment

All patients underwent abdominal ultrasonography to detect the presence of fatty liver (NAFLD). Liver stiffness measurement was performed using ultrasound elastography, and fibrosis staging (F0-F4) was determined based on standard liver stiffness cut-off values.

Outcome measures

The primary outcome was to evaluate the correlation between FIB-4 index and liver stiffness measured by elastography. Secondary outcomes included assessment of the diagnostic accuracy of FIB-4 in detecting significant fibrosis.

Statistical analysis

Data were entered into Microsoft Excel (Microsoft Corporation, Redmond, Washington, USA) and analyzed using appropriate statistical software. Continuous variables were expressed as mean ± standard deviation, and categorical variables as percentages. Correlation between variables was assessed using Pearson or Spearman correlation coefficients, depending on data distribution. Receiver operating characteristic (ROC) curve analysis was performed to evaluate the diagnostic performance of the FIB-4 index. The area under the ROC curve (AUROC) with 95% confidence intervals was calculated. A p-value of <0.05 was considered statistically significant.

## Results

A total of 150 patients with T2DM were included in the study. The baseline demographic, clinical, and biochemical characteristics of the study population are summarized in Table 1. The study population had a mean age of 61.62 ± 11.40 years with a nearly equal gender distribution. The mean BMI was 28.70 ± 6.70 kg/m², and the mean duration of T2DM was 9.32 ± 6.10 years. Basic demographic, glycemic, and biochemical parameters are detailed in Table 1.

**Table 1:** Baseline demographic, clinical, and laboratory characteristics of the study population T2DM: type 2 diabetes mellitus, AST: aspartate aminotransferase, ALT: alanine aminotransferase, FIB-4: Fibrosis-4, NAFLD: non-alcoholic fatty liver disease, HbA1c: hemoglobin A1c, ALP: alkaline phosphatase.

Parameter	Value	Reference Range
Age (years)	61.62 ± 11.40	—
Gender		—
Male	74 (49.3%)	—
Female	76 (50.7%)	—
Body mass index (kg/m²)	28.70 ± 6.70	18.5-24.9 kg/m²
Duration of T2DM (years)	9.32 ± 6.10	—
Random blood sugar (mg/dL)	213.74 ± 45.51	70-140 mg/dL
HbA1c (%)	9.14 ± 1.44	<5.7%
AST (U/L)	59.53 ± 25.30	10-40 U/L
ALT (U/L)	67.11 ± 28.96	7-56 U/L
ALP (U/L)	130.21 ± 42.79	44-147 U/L
Serum albumin (g/dL)	3.75 ± 0.43	3.5-5.0 g/dL
Total cholesterol (mg/dL)	197.06 ± 28.33	<200 mg/dL
Platelet count (/mm³)	229,953.26 ± 75,076.58	150,000-450,000/mm³
FIB-4 score	2.47 ± 1.81	<1.3
NAFLD Fibrosis Score (NFS) [15]	0.19 ± 1.83	<-1.455
Liver stiffness (kPa)	8.97 ± 2.50	<6.0 kPa

Ultrasonographic evaluation demonstrated that the majority of patients with T2DM had NAFLD. The distribution of NAFLD in the study population is presented in Table 2.

**Table 2:** Distribution of NAFLD among patients with T2DM (n = 150) NAFLD: non-alcoholic fatty liver disease, T2DM: type 2 diabetes mellitus.

NAFLD Status	n (%)
No	40 (26.7%)
Yes	110 (73.3%)
Total	150 (100%)

Fibrosis staging based on ultrasound elastography demonstrated that most patients had moderate to advanced fibrosis, with the majority classified in stages F2 and F3. The detailed distribution of fibrosis stages is presented in Table 3.

**Table 3:** Distribution of fibrosis stages based on ultrasound elastography among patients with T2DM evaluated for NAFLD (n = 150) T2DM: type 2 diabetes mellitus, NAFLD: non-alcoholic fatty liver disease.

Stage	n (%)
F0	20 (13.3%)
F1	7 (4.7%)
F2	78 (52.0%)
F3	41 (27.3%)
F4	4 (2.7%)
Total	150 (100%)

The mean FIB-4 score increased progressively with advancing fibrosis stage on ultrasound elastography among patients with T2DM evaluated for NAFLD, with the highest values observed in patients with advanced fibrosis (F3-F4). The distribution of FIB-4 scores across fibrosis stages is summarized in Table 4.

**Table 4:** Distribution of FIB-4 scores according to fibrosis stages on ultrasound elastography among patients with T2DM evaluated for NAFLD (N = 150) FIB-4: Fibrosis-4, T2DM: type 2 diabetes mellitus, NAFLD: non-alcoholic fatty liver disease.

Fibrosis Stage	N	Mean ± SD	95% CI
F0	20	0.85 ± 0.20	0.76-0.94
F1	7	0.88 ± 0.34	0.57-1.19
F2	78	1.73 ± 0.56	1.60-1.86
F3	41	4.49 ± 1.53	4.01-4.97
F4	4	6.98 ± 1.58	4.46-9.50
Total	150	2.47 ± 1.81	2.17-2.76

A strong positive correlation was observed between the FIB-4 score and average liver stiffness measured by ultrasound elastography among patients with T2DM evaluated for NAFLD (Spearman’s ρ = 0.804, 95% CI: 0.737-0.856; p < 0.001). The FIB-4 score demonstrated excellent diagnostic performance for detecting significant fibrosis among patients with T2DM evaluated for NAFLD, with an AUROC of 0.951 (95% CI: 0.918-0.983; p < 0.001). The detailed diagnostic performance is presented in Table 5.

**Table 5:** Diagnostic performance of FIB-4 score for detection of significant fibrosis among patients with T2DM evaluated for NAFLD (N = 150) FIB-4: Fibrosis-4, T2DM: type 2 diabetes mellitus, NAFLD: non-alcoholic fatty liver disease, AUROC: area under the receiver operating characteristic curve.

Parameter	Value	AUROC (95% CI)	Standard Error	p-Value	Overall Model Quality Score
Total sample size (n)	150	0.951 (0.918-0.983)	0.017	<0.001	0.92
Positive cases (significant fibrosis)	123 (82.0%)				
Negative cases	27 (18.0%)				

**Figure 1:**
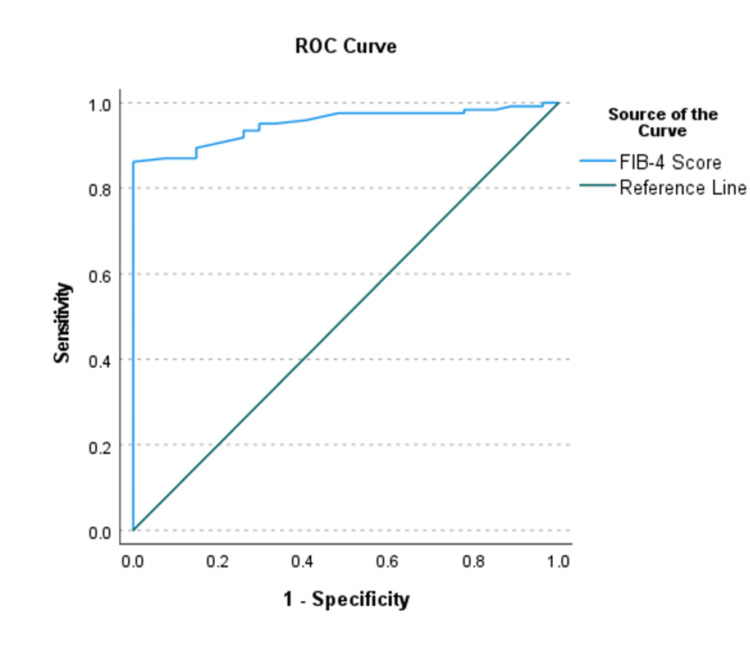
ROC curve showing diagnostic performance of the FIB-4 score for detection of significant fibrosis among patients with T2DM evaluated for NAFLD (N = 150) ROC: receiver operating characteristic, FIB-4: Fibrosis-4, T2DM: type 2 diabetes mellitus, NAFLD: non-alcoholic fatty liver disease.

The ROC curve analysis of the FIB-4 score is illustrated in Figure 1.

Different FIB-4 cut-off values demonstrated varying sensitivity and specificity for the detection of significant fibrosis. A lower cut-off value showed high sensitivity, whereas higher cut-off values demonstrated greater specificity. The diagnostic performance of different FIB-4 cut-off values is summarized in Table 6.

**Table 6:** Diagnostic performance of different FIB-4 cut-off values for detection of significant fibrosis among patients with T2DM evaluated for NAFLD FIB-4: Fibrosis-4, T2DM: type 2 diabetes mellitus, NAFLD: non-alcoholic fatty liver disease, AUROC: area under the receiver operating characteristic curve.

FIB-4 Cut-off	Clinical Purpose	Sensitivity (%)	Specificity (%)
< 0.63	Rule-out threshold (high sensitivity)	98.4	14.8
0.63-1.19	Indeterminate zone	—	—
≥ 1.20	Optimal diagnostic cut-off	87	92.6
≥ 1.23	Rule-in threshold (high specificity)	86.2	100

Comparison of mean FIB-4 scores across fibrosis stages demonstrated statistically significant differences between groups. One-way ANOVA showed an F statistic of 103.47 (df = 4,145; p < 0.001). Similar findings were observed with the Welch test (F = 84.49, df = 415.58; p < 0.001) and Brown-Forsythe test (F = 81.76, df = 49.55; p < 0.001), confirming significant variation in FIB-4 scores across fibrosis stages (Table 7).

**Table 7:** Comparison of mean FIB-4 scores across fibrosis stages based on ultrasound elastography FIB-4: Fibrosis-4.

Test	Statistic	df	p-Value
One-way ANOVA	F = 103.47	4,145	<0.001
Welch Test	F = 84.49	415.58	<0.001
Brown-Forsythe Test	F = 81.76	49.55	<0.001

Post hoc pairwise analysis demonstrated statistically significant differences in mean FIB-4 scores between most fibrosis stages. No statistically significant difference was observed between F0 and F1 stages (p = 1.000) or between F3 and F4 stages (p = 0.225). All other intergroup comparisons showed statistically significant differences in mean FIB-4 scores (Table 8).

**Table 8:** Post hoc pairwise comparison of mean FIB-4 scores across fibrosis stages based on ultrasound elastography FIB-4: Fibrosis-4.

Comparison	Mean Difference	p-Value	Interpretation
F0 vs F1	-0.03	1	Not significant
F0 vs F2	-0.88	<0.001	Significant
F0 vs F3	-3.64	<0.001	Significant
F0 vs F4	-6.13	0.022	Significant
F1 vs F2	-0.85	0.002	Significant
F1 vs F3	-3.61	<0.001	Significant
F1 vs F4	-6.10	0.02	Significant
F2 vs F3	-2.76	<0.001	Significant
F2 vs F4	-5.25	0.034	Significant
F3 vs F4	-2.49	0.225	Not significant

Comparison of laboratory parameters across fibrosis stages demonstrated statistically significant differences for AST, ALT, and platelet count, whereas hemoglobin A1c (HbA1c) did not show a significant difference across fibrosis stages (H = 7.918, p = 0.095). AST levels increased significantly with advancing fibrosis severity (H = 56.494, p < 0.001), while platelet counts progressively decreased across higher fibrosis stages (H = 37.759, p < 0.001). ALT levels also showed significant variation across fibrosis stages (H = 27.701, p < 0.001) (Table 9).

**Table 9:** Comparison of laboratory parameters across fibrosis stages based on ultrasound elastography AST: aspartate aminotransferase, ALT: alanine aminotransferase, HbA1c: hemoglobin A1c.

Variable	F0 (n = 20)	F1 (n = 7)	F2 (n = 78)	F3 (n = 41)	F4 (n = 4)	H (df = 4)	p-Value
HbA1c (%)	8.65 (7.75-9.55)	8.60 (7.80-8.85)	9.35 (7.90-10.70)	8.90 (7.80-9.60)	9.60 (9.15-10.15)	7.918	0.095
	62.5	53.57	83.39	68.72	94.5		
AST (U/L)	35 (29-47.5)	21 (21-43)	51 (37-75)	81 (65-92)	110.5 (99.5-120)	56.494	<0.001
	38.05	31.64	68.6	107.66	144.38		
ALT (U/L)	85.5 (60.5-99.5)	89 (81-95)	64.5 (48-95)	38 (27-72)	96 (92-101.5)	27.701	<0.001
	96.98	97.07	80.21	48.17	118.63		
Platelet count (/mm³)	305,673 (248,238-337,537)	304,456 (234,510-315,906)	257,517 (193,747-295,229)	164,106 (125,537-226,969)	121,870 (109,836-123,195)	37.759	<0.001
	108.45	96.71	81.83	50.07	10.75		

Correlation analysis demonstrated a strong positive association between AST levels and liver stiffness (r = +0.744, p < 0.001). Platelet count showed a moderate-to-strong negative correlation with liver stiffness (r = −0.472, p < 0.001), while ALT demonstrated a moderate negative correlation (r = -0.365, p < 0.001). HbA1c did not show a significant correlation with liver stiffness (r = -0.005, p = 0.954) (Table 10).

**Table 10:** Correlation of laboratory parameters with average liver stiffness on ultrasound elastography AST: aspartate aminotransferase, ALT: alanine aminotransferase, HbA1c: hemoglobin A1c.

Variable	r (95% CI)	p-Value	Strength of Association
HbA1c (%)	-0.005 (-0.170 to 0.160)	0.954	Negligible
AST (U/L)	+0.744 (0.660 to 0.809)	<0.001	Strong positive
ALT (U/L)	-0.365 (-0.500 to -0.213)	<0.001	Moderate negative
Platelet count (/mm³)	-0.472 (-0.591 to -0.333)	<0.001	Moderate-to-strong negative

Correlation analysis between laboratory parameters and FIB-4 score demonstrated a strong positive association with AST levels (r = +0.61, p < 0.001). Platelet count showed a moderate-to-strong negative correlation (r = -0.49, p < 0.001), while ALT demonstrated a moderate negative correlation with FIB-4 score (r = -0.32, p < 0.001). HbA1c did not show a statistically significant association with FIB-4 score (r = +0.04, p = 0.611) (Table 11).

**Table 11:** Correlation of laboratory parameters with FIB-4 score AST: aspartate aminotransferase, ALT: alanine aminotransferase, FIB-4: Fibrosis-4, HbA1c: hemoglobin A1c.

Variable	r (95% CI)	p-Value	Strength of Association
HbA1c (%)	+0.04 (-0.12 to 0.21)	0.611	Not significant
AST (U/L)	+0.61 (0.49 to 0.70)	<0.001	Strong positive
ALT (U/L)	-0.32 (-0.46 to -0.17)	<0.001	Moderate negative
Platelet count (/mm³)	-0.49 (-0.61 to -0.36)	<0.001	Moderate-to-strong negative

A statistically significant association was observed between ultrasound evidence of NAFLD and fibrosis stages on ultrasound elastography (χ² = 73.951, p < 0.001). The strength of association was high, with a Cramer’s V value of 0.702. Most patients with NAFLD were distributed across F2 to F4 fibrosis stages, whereas patients without NAFLD were predominantly in lower fibrosis stages (Table 12).

**Table 12:** Association between ultrasound evidence of NAFLD and fibrosis stages based on ultrasound elastography NAFLD: non-alcoholic fatty liver disease.

Ultrasound Evidence of NAFLD	F0	F1	F2	F3	F4	Total	χ² (df)	p-Value	Cramer's V
Absent (No)	20 (100.0%)	0 (0.0%)	20 (25.6%)	0 (0.0%)	0 (0.0%)	40 (26.7%)	73.951 (4)	<0.001	0.702
Present (Yes)	0 (0.0%)	7 (100.0%)	58 (74.4%)	41 (100.0%)	4 (100.0%)	110 (73.3%)			
Total	20	7	78	41	4	150			

Mean liver stiffness increased progressively with advancing fibrosis stage, ranging from 5.36 ± 0.44 kPa in F0 to 17.30 ± 1.53 kPa in F4. Comparison across fibrosis stages demonstrated statistically significant differences in liver stiffness values on both one-way ANOVA and Welch ANOVA testing (p < 0.001). The effect size was very large (η² = 0.909), indicating a strong influence of fibrosis stage on liver stiffness measurements (Table 13).

**Table 13:** Comparison of average liver stiffness across fibrosis stages based on ultrasound elastography

Fibrosis Stage	n	Mean ± SD (kPa)	95% CI	ANOVA (F, df), p-Value	Welch ANOVA	Effect Size (η² )
F0	20	5.36 ± 0.44	5.15-5.56	F(4, 145) = 360.38, <0.001	F(4, 15.23) = 295.69, p < 0.001	0.909
F1	7	6.61 ± 0.49	6.16-7.07			
F2	78	8.39 ± 0.59	8.26-8.52			
F3	41	11.41 ± 1.08	11.07-11.75			
F4	4	17.30 ± 1.53	14.87-19.73			
Total	150	8.97 ± 2.50	8.56-9.37			

## Discussion

The present study evaluated the utility of the Fibrosis-4 (FIB-4) index and ultrasound elastography for the assessment of liver fibrosis among patients with type 2 diabetes mellitus (T2DM) and demonstrated a high prevalence of non-alcoholic fatty liver disease (NAFLD) and significant fibrosis in this population. NAFLD has emerged as one of the most common chronic liver diseases worldwide and is strongly associated with metabolic syndrome and T2DM [1,2]. Insulin resistance, chronic inflammation, and altered lipid metabolism are considered major contributors to the development and progression of NAFLD in patients with diabetes [3,6].

In the present study, 73.3% of patients with T2DM had evidence of NAFLD on ultrasonography. This finding is consistent with previously published studies demonstrating a high prevalence of NAFLD among diabetic individuals [1,8]. Williamson et al. reported a similarly high burden of hepatic steatosis among patients with T2DM, emphasizing the close relationship between diabetes and fatty liver disease [8]. The high prevalence observed in the current study further supports recommendations for routine screening for NAFLD in patients with T2DM [2].

A significant proportion of patients in the present study demonstrated moderate to advanced fibrosis, with most patients classified in fibrosis stages F2 and F3 on ultrasound elastography. Previous studies have shown that fibrosis stage is the most important histological predictor of liver-related morbidity and mortality in NAFLD [4,5]. Advanced fibrosis has also been associated with increased cardiovascular and extrahepatic complications [7,10]. Therefore, early identification of fibrosis among patients with T2DM is clinically important to reduce long-term complications and improve outcomes.

The mean FIB-4 score increased progressively with advancing fibrosis stage, and a strong positive correlation was observed between FIB-4 score and liver stiffness measured by ultrasound elastography (Spearman’s ρ = 0.804, p < 0.001). These findings indicate that higher FIB-4 scores are associated with increasing fibrosis severity. Similar observations have been reported in previous studies evaluating non-invasive fibrosis markers in NAFLD [12,13]. Sterling et al. originally developed the FIB-4 index as a simple non-invasive marker for fibrosis assessment using routinely available laboratory parameters [12]. Subsequently, Shah et al. demonstrated the usefulness of FIB-4 in identifying advanced fibrosis in patients with NAFLD [13].

In the present study, the FIB-4 score demonstrated excellent diagnostic accuracy for detection of significant fibrosis, with an AUROC of 0.951. This finding suggests that the FIB-4 index may serve as an effective screening tool in patients with T2DM and NAFLD. The evaluated FIB-4 cut-off values also demonstrated clinically useful diagnostic performance. A lower cut-off value showed very high sensitivity and may help exclude significant fibrosis, whereas higher cut-off values demonstrated excellent specificity for identifying advanced fibrosis. These findings are clinically relevant because the FIB-4 index is inexpensive, easily calculable, and widely accessible in routine clinical practice.

Ultrasound elastography demonstrated a progressive increase in liver stiffness values across fibrosis stages, with highly significant differences between groups. Previous studies have shown that liver stiffness measurement correlates well with hepatic fibrosis severity and can be used as a reliable non-invasive alternative to liver biopsy [14]. Although liver biopsy remains the gold standard for fibrosis assessment, its use is limited by invasiveness, sampling variability, cost, and procedure-related complications [11]. Therefore, non-invasive approaches combining serum fibrosis markers and elastography may provide practical alternatives for fibrosis screening and risk stratification.

The present study also demonstrated significant associations between fibrosis severity and laboratory parameters. AST levels showed a strong positive correlation with liver stiffness and FIB-4 score, whereas platelet count showed a moderate-to-strong negative correlation. Progressive thrombocytopenia in advanced fibrosis may be explained by portal hypertension and splenic sequestration associated with chronic liver disease [16]. In contrast, HbA1c did not show a statistically significant association with fibrosis severity or liver stiffness in the present study. A statistically significant association was observed between ultrasound evidence of NAFLD and fibrosis stage, with most patients with NAFLD distributed across higher fibrosis stages. These findings further support the progressive nature of NAFLD in patients with T2DM and emphasize the need for early fibrosis assessment in this high-risk population.

The present study has certain limitations. Being a single-center cross-sectional study, causal relationships could not be established. Additionally, liver biopsy was not performed for histopathological confirmation of fibrosis. However, the study provides valuable evidence regarding the role of non-invasive fibrosis assessment tools in patients with T2DM and NAFLD. The relatively adequate sample size and comprehensive evaluation using both biochemical and imaging-based modalities strengthen the findings of the study.

## Conclusions

The present study demonstrates a high prevalence of non-alcoholic fatty liver disease and significant liver fibrosis among patients with type 2 diabetes mellitus, highlighting the need for early detection in this high-risk population. A substantial proportion of patients exhibited moderate to advanced fibrosis, often without clear clinical symptoms. The Fibrosis-4 (FIB-4) index showed a strong correlation with liver stiffness measured by ultrasound elastography and demonstrated excellent diagnostic accuracy in identifying significant fibrosis. Given its simplicity, cost-effectiveness, and reliance on routinely available laboratory parameters, FIB-4 represents a practical non-invasive screening tool. Its incorporation into routine clinical practice may facilitate early risk stratification, guide further evaluation, and support timely clinical intervention, thereby potentially reducing the burden of advanced liver disease in patients with type 2 diabetes mellitus.
